# Best of United European Gastroenterology Week 2025

**DOI:** 10.1002/ueg2.70171

**Published:** 2026-01-28

**Authors:** Iuliana Nenu, Sara Nikolic, Eduardo Valdivielso, Giovanni Marasco

**Affiliations:** ^1^ Department of Functional Sciences, Physiology Discipline “Iuliu Haţieganu” University of Medicine and Pharmacy Cluj‐Napoca Romania; ^2^ Department of Gastroenterology University Medical Center Maribor Maribor Slovenia; ^3^ Department of Gastroenterology A Coruña Universitary Hospitalary Complex (CHUAC) A Coruña Spain; ^4^ Department of Medical and Surgical Sciences University of Bologna Bologna Italy

## 1 Introduction

UEG Week 2025, held in Berlin, brought together over 12,200 participants from 115 countries, including more than 5200 postgraduate trainees, and showcased 4168 abstracts in Gastroenterology and Hepatology, spanning basic science to clinical practice. The week opened with an inspiring address by *UEG President Prof. Dr. Mathias Löhr* and *Scientific Committee Chair Prof. Dr. Julia Mayerle*, setting the tone for an engaging and exciting forum for the international community to exchange innovative ideas, research, and practice‐changing clinical insights.

A major novelty at UEG Week 2025 was the debut of the *Digestive Disease Mechanism (DDM) Summit*. This 3‐day standalone program on gastrointestinal pathogenesis, including malignancies, highlighted the fundamental role of basic science in translational gastroenterology. The summit was exceptionally well‐organized and drew outstanding interest from basic researchers around the world, providing a high‐quality platform for scientific discussion. We are pleased to announce that the DDM Summit will take place again in Barcelona in 2026 alongside UEG Week.

The purpose of this editorial is to share the key highlights and pivotal takeaways from UEG Week 2025 with the academic community. The authors have selected works based on their scientific impact, recognition by the scientific committee and their direct relevance to the clinical themes discussed in the following section. To achieve this clarity, we have structured this summary into distinct sections focusing on Oncology, Endoscopy, Inflammatory Bowel Diseases, Luminal and Neurogastroenterology and Motility, Pancreatology, and Hepatology.

### 1.1 Oncology Highlights

Two oncology studies were recognized among the best abstracts. In the multicentre ENDURO randomized trial, endoscopic ultrasonography‐guided gastroenterostomy (EUS‐GE) proved superior to surgical gastroenterostomy (SGJ) for palliative management of malignant gastric outlet obstruction. EUS‐GE allowed faster return to solid oral intake (median 1 vs. 3 days; HR 2.21, *p* = 0.0003) and shorter hospital stay (1 vs. 4 days), while remaining non‐inferior regarding recurrent obstruction (10% vs. 12%) and achieving higher clinical success (96% vs. 80%) with comparable safety, positioning EUS‐GE as the preferred palliative approach.

*Kenneth Peuker* demonstrated that calcineurin activity in T cells is essential for effective anti‐tumor immunity across multiple solid tumor types. By sustaining early CD8+ T cell activation and proliferation through one‐carbon metabolism and nucleotide biosynthesis, calcineurin supports tumor infiltration and cytotoxic function. These findings uncover a metabolic hub linking immunosuppression to increased cancer risk, providing mechanistic insight into how calcineurin inhibitors may promote tumor progression in patients receiving immunosuppressive therapy.

### 1.2 Endoscopy Highlights

Interventional EUS has evolved considerably in recent years, as reflected in the studies presented at UEG Week. Several abstracts highlighted the good results of EUS‐guided choledocoduodenostomy (EUS‐CDS) in draining distal malignant biliary strictures, rivalling ERCP while offering a lower incidence of post‐procedural pancreatitis (*Jeska Fritzsche; Marco Spadaccini*). In addition to already mentioned ENDURO trial, The LONG‐RANGE trial presented by *Giuseppe Vanella* further supported the efficacy of EUS‐GE, reporting a low dysfunction rate (6.4%) and effective endoscopic reintervention; these findings suggest that routine endoscopic surveillance may be unnecessary in malignant gastric outlet obstruction (GOO), and that LAMS exchange or removal every 8–12 months may suffice in benign GOO.

*Nik Dekkers* reported that Endoscopic Submucosal Dissection (ESD) achieves lower local recurrence, higher R0 resection rates, shorter hospitalization, reduced costs, improved functional outcomes, and a comparable safety profile relative to Transanal Minimally Invasive Surgery (TAMIS) for rectal lesions (TRIASSIC study). Furthermore, the adoption of underwater or saline immersion techniques in both ESD and EMR seems to facilitate safer, faster, and technically easier resections. There is a growing shift toward cold snare resection, which offers improved safety compared to hot snare techniques, albeit with a slightly higher recurrence rate after adenoma removal, as shown in the abstract presented by Federico A Peralta IV et al.

Regarding artificial intelligence (AI), studies show that computer‐aided detection (CADe) does not increase adenoma detection rate (ADR) when baseline ADR is high, but enhances serrated lesion detection—except among highly experienced endoscopists—without prolonging withdrawal time or increasing unnecessary polypectomies (Project CAD, presented by *Sekiguchi Masau*). Moreover, autonomous computer‐aided diagnosis (CADx) systems have proven feasible in real‐world settings, fulfilling criteria for “resect‐and‐discard” and “diagnose‐and‐leave” strategies, achieving high accuracy, robust rectosigmoid NPV, and strong patient acceptance.

Finally, the ESGE allowed us to observe how the endoscopy stars perform complex procedures, as well as to practice techniques in the Hands‐On Workshops with renowned teachers.

### 1.3 Inflammatory Bowel Diseases Highlights

The program was exceptionally rich and diverse in IBD, making it challenging to spotlight only a few studies. The plenary opening session set the tone, with *Mette Julsgard* presenting a groundbreaking study on JAK inhibitor (JAKi) exposure during pregnancy in women with IBD. Among 47 exposures, 35 pregnancies progressed to term, maternal complication rates were low, and no increased risk of adverse outcomes was observed in live births. Although disease activity was common—especially after early discontinuation of JAKi—infant outcomes, including development, infection risk, and vaccine response, were reassuring.

In contrast, the GETAID CURE study, presented by *Bénédicte Caron*, investigated withdrawal of advanced therapy in early Crohn's disease in a prospective manner. Among the 22.6% of patients who achieved deep remission at 1 year on adalimumab, only about a quarter maintained deep remission at 2 years. These results suggest that anti‐TNF therapy should not be discontinued even in early‐stage Crohn's disease.

Offering a more optimistic perspective, the BEST‐CD randomized controlled trial from India presented by *Partha Pal* shared with us interim results showing that endoscopic suturing significantly reduced clinical recurrence, re‐intervention, and hospitalizations compared with endoscopic balloon dilatation for short Crohn's‐related strictures, while maintaining similar safety profiles.

Although endoscopy remains irreplaceable for direct visualization of mucosal lesions and for histological assessment, intestinal ultrasound continues to gain momentum due to its non‐invasiveness, repeatability, and reduced environmental impact. This was reflected at UEGW, where an entire session was dedicated to advances in intestinal ultrasound technology and applications.

Looking toward the future, *Bruce Sands* discussed obefazimod—an oral, once‐daily small molecule that enhances expression of microRNA‐124. Studied in Phase 3 trial in moderately to severely active ulcerative colitis, obefazimod produced significant improvements in clinical, endoscopic, symptomatic, and combined endoscopic‐histologic endpoints by week 8. It was well tolerated with no new safety signals identified. Meanwhile, *Silvio Danese* introduced emerging anti‐TL1A inhibitors, still in early Phase 2 development. These agents—afimkibart, duvakitug, and tulikisobart—target and block the TL1A protein, a key driver of inflammation and fibrosis. All three showed promising results, achieving primary and secondary outcomes in both ulcerative colitis and Crohn's disease.

### 1.4 Luminal and Neurogastroenterology and Motility Highlights

The 2025 UEG Week showcased substantial progress across luminal digestive diseases and neurogastroenterology. Within disorders of gut–brain interaction (DGBI), **Claudia Barber** and colleagues demonstrated that a simple non‐instrumental behavioral technique correcting abdominophrenic dyssynergia achieved a 24% reduction in abdominal distension versus 8% with placebo, with sustained improvement at 3 months (≈37% reduction in distension and 34% in bloating). These data confirm that targeted diaphragmatic retraining offers durable symptom relief without instrumentation. In parallel, *Tom van Gils* analyzed 5448 participants, showing that bloating and visible distension are distinct yet overlapping features: 64.9% of irritable bowel syndrome (IBS), 50.6% of functional dyspepsia (FD), and 88.5% of overlapping IBS + FD patients reported either symptom, compared with 13.7% in controls. Dietary interventions were also central in DGBI research. In the *MED‐IBS Trial*, 139 patients were randomized to a Mediterranean diet or traditional dietary advice (TDA) for 6 weeks. The primary clinical response (≥ 50‐point IBS‐SSS reduction) occurred in 62% of the Mediterranean‐diet group versus 42% with TDA, confirming the Mediterranean pattern as a superior first‐line option for the treatment of IBS patients. Parallel mechanistic insight came from *Arnau Vich Vila* et al. in the *DOMINO study*, analyzing 1700 samples from 463 patients. Participants with baseline dysbiosis were less likely to respond to FODMAP restriction, while moderate restriction preserved *Bifidobacterium* abundance compared with strict regimens, suggesting that partial dietary reduction optimizes efficacy without compromising microbial health.

As for *Helicobacter pylori* infection**,** *Imane Kirrout* presented the *Hp‐EuReg analysis* of 18,219 patients from 13 countries. Despite multiple statistically significant correlations, antibiotic‐resistance rates derived from the literature showed minimal predictive value for empirical *Helicobacter pylori* eradication (e.g., clarithromycin OR 1.02 [1.00–1.03]; levofloxacin OR 0.98 [0.97–1.00]), reinforcing the need for local surveillance rather than reliance on pooled estimates.

Major advances were also seen in eosinophilic esophagitis (EoE). From the *EoE CONNECT Registry*, *Luis Rodríguez‐Alcolado* analyzed 3900 patients, showing that concomitant atopy significantly lowered PPI response rates—42.7% versus 49.7% (asthma) and 39.3% versus 48% (urticaria)—while multiple atopic comorbidities further reduced efficacy. In a multicentre real‐world study enrolling 292 patients, *Daria Maniero* reported that orodispersible budesonide induced histologic remission in 93% and endoscopic remission in 53% of patients after 12 weeks, maintaining histologic response in 92% at one year with a favorable safety profile (candidiasis 12%). Finally, the pediatric *EoE KIDS Phase III Trial*, presented by *Margaret H. Collins*, demonstrated that dupilumab reduced peak eosinophil counts by −86% versus +21% with placebo (*p* < 0.001) and maintained architectural and inflammatory improvement at 52 weeks, confirming durable biologic efficacy.

In celiac disease, *Alina Pes* **i** and *Detlef Schuppan* presented the *CEC‐3* and *CEC‐004/CEL trials* on the TG2 inhibitor ZED1227. The CEC‐3 analysis showed upregulation of 93% of the top 100 regulatory genes under ZED1227 versus placebo, indicating robust activation of T‐regulatory pathways. In CEC‐004, 397 patients were randomized; although the composite histology + symptom endpoint was not met, the 50 mg QD dose achieved a significant villous‐height‐to‐crypt‐depth improvement over placebo with good tolerability. In contrast, *Luca Elli* reported that the *TAK‐062 phase II trial* failed to show symptom or histologic benefit but contributed key mechanistic insights for next‐generation glutenase design.

### 1.5 Pancreatology Highlights

At this year's conference, *Christoph Ammer‐Hermanau* once again underscored the predictive power of the orointestinal microbiome in the natural course of acute pancreatitis. The MAMBA trial demonstrated that microbial sequencing obtained during the initial emergency room phase of acute pancreatitis is strongly associated with post‐discharge diabetes, recurrent acute pancreatitis, and mortality — suggesting that early microbiome profiling may hold valuable prognostic potential.

*Enrique de Madaria*, recipient of last year's research prize, presented results from the WATERLAND trial, the largest and most globally representative randomized clinical study ever conducted in acute pancreatitis. The trial confirmed that Lactated Ringer's solution exerts a consistent anti‐inflammatory effect and is linked to a lower incidence of acidosis and hyperchloremic acidosis. However, these physiological benefits did not translate into improvements in major clinical outcomes such as symptom severity, disease progression, complication rates, hospital stay duration, or mortality.

From the same research group, *Lucia Guilabert* reported that simvastatin does not appear to protect against recurrent episodes of acute pancreatitis or acute‐on‐chronic pancreatitis.

The UEG Journal session, held in vibrant “pancreas colors,” explored several cutting‐edge topics: the management of splanchnic thrombosis in pancreatic diseases, evidence‐based strategies for treating walled‐off necrosis following initial drainage, and the emerging role of AI in pancreatology. *Malte Buchholz* received the Best Paper Award for his team's groundbreaking work. His group developed a serum‐based multianalyte test that combines a 7‐gene mRNA/miRNA signature with CA 19‐9 to detect recurrence after resection of pancreatic ductal adenocarcinoma. The combined panel achieved 90% diagnostic accuracy (98% sensitivity, 84% specificity), outperforming CA 19‐9 alone. Impressively, it may also identify recurrence earlier than standard follow‐up, offering a noninvasive tool for timely postoperative monitoring and intervention [1].

Finally, the conference featured 167 pancreas‐related poster presentations, all of which—along with the sessions mentioned above—are available for viewing on Gutflix.

### 1.6 Hepatology Highlights

Among the top‐ranked abstracts in hepatology, the study by *Andreas Kremer* presented particularly impactful data. In the Phase 3 GLISTEN study, the ileal bile acid transporter inhibitor linerixibat (40 mg twice daily) provided a rapid and clinically meaningful improvement in pruritus among patients with primary biliary cholangitis, with a mean reduction in itch intensity of −2.86 versus −2.15 for placebo (*p* = 0.001), and an early benefit evident by Week 2 (*p* < 0.001). Linerixibat also significantly improved sleep interference (*p* = 0.024), with 41% of patients achieving *a* ≥ 4‐point itch reduction versus 29% on placebo. Gastrointestinal adverse events, mainly diarrhea (61%), were more common but generally mild, leading to discontinuation in only 4% of patients.

*Lihe Liu* reported that both sugar‐sweetened and low‐ or non‐sugar‐sweetened beverages were associated with an increased risk of metabolic dysfunction–associated steatotic liver disease (MASLD), with artificially sweetened drinks additionally linked to higher liver‐related mortality. Substituting these beverages with water appeared to mitigate this risk. Data from ESSENCE Phase 3 trial was presented revealing that Semaglutide 2.4 mg significantly improved metabolic dysfunction‐associated steatohepatitis (MASH)‐related histological and non‐invasive endpoints, as well as fibrosis‐related NITs, with approximately half of its therapeutic effect occurring independently of weight loss. These findings suggest that semaglutide exerts substantial weight loss‐independent metabolic actions that contribute meaningfully to MASH resolution and fibrosis improvement. In addition, *Oriol Juanola* received a Best Presentation Award for a study on the role of Paneth cells in MASLD pathogenesis.

Also, regarding the Hepatology topic, the UEG Research Prize 2025 was awarded to *Fotios Sampaziotis* for advancing regenerative cell therapy in liver transplantation. Using human biliary organoids to repair ischemic bile ducts ex vivo, this approach could reduce the number of livers discarded after circulatory death and improve patient outcomes.

### 1.7 Guidelines, Hands‐On, Social Media and Networking

“Guidelines” and “consensus” remain favorite words among gastroenterologists — and this year did not disappoint. Several important documents were published in the *UEG Journal* ahead of UEG Week, including the *Consensus on Functional Bloating and Abdominal Distension,* new guidelines on *malabsorption*, and an *updated guideline for celiac disease* [2, 3, 4, 5]. During UEG Week itself, it was announced that the guidelines for pain management in acute pancreatitis are nearing completion. Updates were also shared on the global evidence‐based guidelines for pancreatic cystic neoplasms and on recommendations for proton pump inhibitor (PPI) prescription. In addition, *Patrick Van Rheenen* presented the new pediatric guidelines for ulcerative colitis, rounding off what has been a particularly productive year for evidence‐based practice in gastroenterology.

Beyond the high‐level research, UEG Week 2025 reinforced its commitment to practical clinical education. Besides well known ESGE workshops, a highly successful aspect noted by participants was the series of abdominal ultrasound “hands‐on” workshops.

The conference began with a captivating lecture by *Wendi Le Brett* titled “How to Fight Fake News in Medicine?” She highlighted that since the COVID‐19 pandemic, social media use has risen across all age groups, with 59% of adults in Europe now turning to social platforms for health information — the highest rates seen in Denmark, Cyprus, and Hungary. As Le Brett put it, “even if you don't use social media, it finds you.” In the United States, she noted, social media nearly rivals physicians as a primary source of medical information — particularly in areas such as irritable bowel syndrome. Her message to clinicians was clear: be brave, get online, and share accurate medical knowledge. Through practical examples and genuine encouragement, Wendi Le Brett's talk stood out as both inspiring and empowering, reflecting her deep commitment to combating misinformation in medicine.

Session recordings and additional materials from UEGW 2025 are available on Gutflix, the official UEG educational platform, for those who were unable to attend the congress and for participants who wished to follow two sessions simultaneously.

As every year, UEG once again hosted an unforgettable closing party. This time, participants were transported to 1920s Germany — a world of cabaret, swing, pearls, and long silk gloves. A memorable finale to an exceptional week!

## 2 Conclusion

The research presented at UEG Week is made possible by the thousands of patients who participate in the trials and studies presented. While this editorial covers only a fraction of the significant work showcased in Berlin, the innovation found in every session is vital to our field. We also encourage young gastroenterologists to join this thriving community and contribute with their work and passion. We look forward to building on these insights at UEG Week 2026 in Barcelona. Hasta pronto!

## Conflicts of Interest

The authors declare no conflicts of interest.

## Data Availability

The authors have nothing to report.
